# De-escalation of antifungal treatment in critically ill patients with suspected invasive *Candida* infection: incidence, associated factors, and safety

**DOI:** 10.1186/s13613-018-0392-8

**Published:** 2018-04-19

**Authors:** Karim Jaffal, Julien Poissy, Anahita Rouze, Sébastien Preau, Boualem Sendid, Marjorie Cornu, Saad Nseir

**Affiliations:** 1 Critical Care Center, CHU Lille, 59000 Lille, France; 2 U995-LIRIC-Lille Inflammation Research International Center, Univ. Lille, 59000 Lille, France; 3 Inserm, U995, 59000 Lille, France; 4 Laboratory of Mycology and Parasitology, CHU Lille, 59000 Lille, France

## Abstract

**Background:**

Antifungal treatment is common in critically ill patients, but only a small proportion of patients receiving antifungals have a proven fungal infection. However, antifungal treatment has side effects such as toxicity, emergence of resistance, and high cost. Moreover, empirical antifungal treatment is still a matter for debate in these patients. Our study aimed to determine the incidence, associated factors, and safety of de-escalation of antifungals in critically ill patients.

**Methods:**

This retrospective study was conducted in a 30-bed mixed ICU, from January 2012 through January 2013. Patients hospitalized for > 5 days and treated with antifungals for first suspected or proven invasive *Candida* infection were included. Exclusion criteria were prophylactic antifungals, suspected invasive aspergillosis, and neutropenia. De-escalation was defined as switch from initial systemic antifungals (except fluconazole) to triazoles, or stopping initial drugs within the 5 days following their initiation.

**Results:**

One hundred and ninety patients were included. Antifungal treatment was empirical, preemptive, and targeted in 55, 27, and 24% of study patients, respectively. Caspofungin (53%), fluconazole (43%), voriconazole (4%), and liposomal amphotericin B (0.5%) were the more frequently used antifungals. De-escalation was performed in 38 (20%) patients. Invasive mechanical ventilation was independently associated with lower rates of de-escalation (OR 0.25 [95% CI 0.08–0.85], *p *= 0.013). Total duration of antifungal treatment was significantly shorter in patients with de-escalation, compared with those with no de-escalation (med [IQR] 6 (5, 18) vs. 13 days (7, 25), *p *= 0.023). No significant difference was found in duration of mechanical ventilation (22 [5–31] vs. 20 days [10–35], *p *= 0.43), length of ICU stay (25 [14–40) vs. 25 days [11–40], *p *= 0.99), ICU mortality (45 vs. 59%, *p *= 0.13), or 1-year mortality (55 vs. 64%, *p *= 0.33) between patients with de-escalation and those with no de-escalation, respectively.

**Conclusions:**

De-escalation was performed in 20% of patients receiving systemic antifungals for suspected or proven invasive *Candida* infection. Mechanical ventilation was independently associated with lower rates of de-escalation. De-escalation of antifungal treatment seems to be safe in critically ill patients.

## Background

Invasive fungal infections are common in critically ill patients [1–3]. Candidiasis is the most frequent fungal infection in hospitalized patients worldwide [4]. Despite a frequency twice less important than the frequency of bacteremia, mortality linked to candidemia is twice higher than that linked to bacteremia [5]. In case of septic shock, this mortality can reach 60% [6]. Despite the introduction of several extended-spectrum triazoles and echinocandin antifungal agents with superior safety, spectrum, and potency, the incidence of invasive *Candida* infection and the associated mortality have not decreased over the past two decades [2]. The high mortality rate is related to comorbidities and also to the difficulty in diagnosis coupled with challenges in prompt adequate antifungal therapy [7].

Clinical signs of invasive *Candida* infection are nonspecific, risk factors are common, the predictive positive value of all scores set to help clinicians remains insufficient [8, 9], and blood cultures have insufficient diagnostic accuracy [10, 11]. Because prompt antifungal treatment has a major impact on mortality [6, 12], guidelines recommend initiating systemic antifungal therapy, for critically ill patients with risk factors for invasive *Candida* infection and no other known cause for fever [13]. Criteria for initiating such therapy in clinical practice remain poorly defined. Consequently, the lack of rapid, sensitive, and specific diagnostic tests can lead to possible overuse of antifungal agents without further confirmation of invasive *Candida* infection [14]. Moreover, the overuse of antifungal agents is associated with increased prevalence of *Candida* non-albicans species and antifungal resistance [15–18]. Other potential consequences of inappropriate use of antifungals are increased cost, drug toxicity, and adverse drug interactions [19, 20].

A cross-sectional multicenter study showed that antifungal treatment was administered to 7.5% of ICU patients, although two-thirds of them had no documented invasive candidiasis [14]. In addition, recent studies suggested no benefit of empirical antifungal treatment in these patients [21, 22]. Reducing antifungal use in the ICU with an antifungal stewardship is feasible and would allow avoiding drawbacks. The European Society for Clinical Microbiology and Infectious Diseases (ESCMID) and the Infectious Diseases Society of America (IDSA) guidelines recommend a de-escalation strategy (5 days in stabilized patients for the IDSA and 10 days overall for the ESCMID) [13, 23], but the level of recommendations is low. The safety of de-escalation in the case of proven invasive *Candida* infection has been suggested by prospective recent studies [24–26]. However, some limitations, such as non-comparative or post hoc design, preclude definite conclusions. Therefore, we hypothesized that de-escalation of antifungal treatment might be safe in patients with suspected invasive *Candida* infection and conducted this retrospective study to identify the incidence and associated factors, and to assess safety of antifungal treatment de-escalation in ICU patients.

## Methods

### Study design

This retrospective observational study was performed in a 30-bed mixed ICU, located in the University Hospital of Lille, France. All data were retrospectively collected during a one-year period (from January 2012 through January 2013). The study was approved by the local Institutional Review Board (Comité de Protection des Personnes Nord-ouest IV). Because of the retrospective observational design of the study, and in accordance with the French law, written informed consent was not required by the local IRB.

### Definitions and studied population

De-escalation of antifungal treatment was defined as either a switch from initial antifungals, except fluconazole, to triazoles, or discontinuation of initial antifungal treatment within the 5 days following their initiation [26]. Proven and suspected fungal infections were defined according to the revised criteria of the European Organization for Research and Treatment of invasive fungal infections Cooperative Group (EORTC) [27]. All patients hospitalized for more than 5 days and requiring systemic antifungal treatment for the first documented or suspected invasive *Candida* infection during their ICU stay were eligible. Patients receiving prophylactic antifungal treatment were excluded, as well as those with suspected mold infection, or neutropenia.

### Study objectives

The primary objective was to evaluate the factors independently associated with antifungal de-escalation. Secondary objectives were to evaluate the incidence of de-escalation of antifungal treatment, and its impact on ICU length of stay, duration of mechanical ventilation, and mortality.

### Data collection

All data were retrospectively recorded from archived medical records of University Hospital of Lille and its mycology laboratory. Patients were identified using the electronic pharmacy database setup to guide and monitor antifungal prescriptions. Only first episodes of proven or suspected invasive *Candida* infection were considered. Initial antifungal treatment was based on local guidelines, driven from international guidelines [28].

The following characteristics were recorded at ICU admission: age, gender, severity of acute illness based on simplified acute physiology score (SAPS) II, comorbidities (diabetes, chronic obstructive pulmonary disease (COPD), chronic heart failure, cirrhosis, chronic renal failure requiring dialysis, or immunosuppression), location before ICU admission, admission category (medical or surgical), reason for ICU admission (acute exacerbation of COPD, acute respiratory distress syndrome, pneumonia, congestive heart failure, neurologic failure, poisoning, shock, and infection), and prior antibiotic or antifungal treatment used in the last 3 months. During ICU stay, data were collected on type of antifungal treatment (empirical, preemptive, or curative), suspected (probable and possible categories of the EORTC definitions) or proven invasive *Candida* infection, successive antifungal treatments prescribed, duration of each antifungal treatment, total duration of antifungal therapy, and appropriateness of the initial antifungal treatment. The following data were collected regarding de-escalation: date of onset and reasons for de-escalation. We also collected the results of mycological cultures from sterile sites (blood, cerebrospinal, pleural, peritoneal and pericardial fluid, surgical site) and the sites usually checked for the multifocal colonization status (skin, urine, trachea, catheter, anus), and data on antibiotic treatment, severe sepsis, total parenteral nutrition, surgery, renal replacement therapy, length of mechanical ventilation, duration of treatment with vasoactive drugs, length of ICU stay, occurrence of apyrexia, ICU, and 30-day and 1-year mortality.

### Statistical analysis

SPSS software (SPSS, Chicago, IL, USA) was used for data analysis. Categorical variables were described as frequency (%). Kolmogorov–Smirnov test was used to evaluate the distribution of continuous variables. Normally distributed and skewed continuous variables were described as mean ± standard deviation (SD), or as median and interquartile range (IQR), respectively.

To determine factors associated with de-escalation, patients with de-escalation were compared with those with no de-escalation using univariate and multivariate analyses. Student’s *t* test or the Mann–Whitney *U* test was used to compare normally distributed and skewed continuous variables, respectively. The Chi-square (χ^2^) test or Fischer’s exact test was used to compare qualitative variables, as appropriate. The odds ratio (OR) and 95% confidence interval (CI) were calculated for all qualitative variables significant in univariate analysis and for all variables significant in multivariate analysis. Differences were considered significant if *p* values were < 0.05, with two-tailed tests. Exposure to potential factors associated with antifungal de-escalation was considered until the occurrence of de-escalation, or until ICU discharge in patients with no de-escalation. All variables with *p* values < 0.2 by univariate analysis were included in a backward multivariate logistic regression model. Potential interactions were tested, and the Hosmer–Lemeshow goodness of fit and *c*-statistics were calculated. Cox proportional hazards models were performed to determine factors associated with ICU mortality.

## Results

**Fig. 1:**
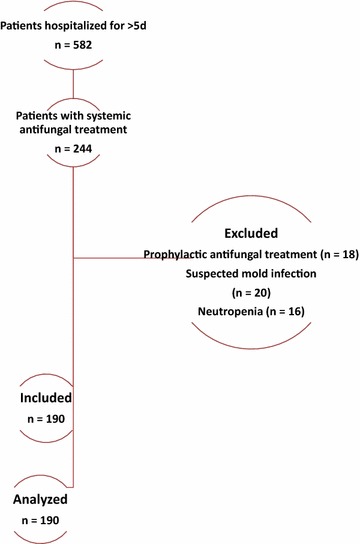
Patient flowchart

Among the 582 patients hospitalized for > 5 days during the study period, 244 (42%) patients received antifungal treatment. Fifty-four (22%) patients were excluded, because they received prophylactic antifungal treatment (*n *= 18, 8%), had suspected filamentous fungal infection (*n *= 20, 8%), or were neutropenic (*n *= 16, 7%). The remaining 190 patients were all included in the study (Fig. 1). One hundred and five (55%), 52 (27%), and 46 (24%) patients received empirical, preemptive, and targeted treatment, respectively. Caspofungin (*n *= 101, 53%), fluconazole (*n *= 81, 43%), voriconazole (*n *= 7, 4%), and liposomal amphotericin B (*n *= 1, 0.5%) were the most frequently used antifungals.

### Patient characteristics and incidence of de-escalation

De-escalation was performed in 38 (20%) of the 190 included patients. Initial antifungal treatment was stopped in 19 (50%) patients within 5 days and switched to an azole in 19 (50%) patients. Reasons for de-escalation were susceptible strain based on antifungal susceptibility testing in 16 (42%) patients, proven bacterial infection with no evidence for fungal infection in 10 (26%) patients, and negative mycological investigations in 12 (32%) patients.

Patient characteristics are presented in Table 1.

**Table 1:** Characteristics of study patients at ICU admission

Characteristics	De-escalation		*p*
	Yes (*n *= 38)	No (*n *= 152)	
Age, years	63 [56–68]	63 [55–72]	0.57
Female gender *n* (%)	8 (21)	55 (36)	0.067
SAPS II	49 [30–68]	54 [36–71]	0.38
Comorbidities *n* (%)			
Diabetes	8 (21)	30 (20)	0.87
COPD	9 (24)	33 (22)	0.79
Chronic heart failure	8 (21)	30 (20)	0.87
Cirrhosis	4 (11)	10 (6)	0.49
Chronic dialysis	9 (24)	14 (9)	0.014*
Immunosuppression *n* (%)			
Chemotherapy	7 (18)	13 (9)	0.076
Corticosteroid therapy	9 (24)	29 (19)	0.53
Transfer from			0.66
Home	4 (11)	21 (14)	
Other wards	25 (66)	104 (68)	
Other ICUs	9 (24)	27 (18)	
Admission category			0.75
Medical	22 (58)	98 (64)	
Surgical	15 (39)	51 (34)	
Other (trauma, burn)	1 (3)	3 (2)	
Cause for ICU admission			
Acute exacerbation of COPD	3 (8)	26 (17)	0.16
Acute respiratory distress syndrome	12 (32)	41 (27)	0.57
Community-acquired pneumonia	11 (29)	32 (21)	0.30
Hospital-acquired pneumonia	6 (16)	38 (25)	0.23
Congestive heart failure	0 (0)	7 (5)	0.18
Neurologic failure	0 (0)	7 (5)	0.18
Poisoning	1 (3)	15 (10)	0.15
Septic shock	22 (58)	89 (59)	0.94
Infection at ICU admission	37 (97)	135 (89)	0.17
Prior antibiotic treatment	16 (42)	69 (45)	0.72
Prior antifungal treatment	5 (13)	15 (10)	0.56

Data are N (%), or median (interquartile range)

*COPD* chronic obstructive pulmonary disease, *ICU* intensive care unit, *SAPS* simplified acute physiology score

* Odds ratio (95% confidence interval) 3.1 (1.21–7.74)

### Mycological results

Thirty-four (18%) of the 187 samples taken from sterile sites were positive, of which 26 (76%) were positive to *Candida albicans*, 2 (6%) to *Candida glabrata*, and 6 (18%) to other *Candida* species. Of 192 samples taken from non-sterile sites, 170 (89%) samples were positive, including 99 (58%) to *C. albicans*, 27 (16%) to *C. parapsilosis*, 20 (12%) to *C. glabrata*, 17 (10%) to *C. tropicalis*, and 7 (4%) to other *Candida* species.

### Factors associated with antifungal de-escalation

By univariate analysis, factors associated with higher rate of de-escalation were chronic dialysis, negative mycological samples, proven bacterial infection, apyrexia for > 72 h, and vasoactive drug discontinuation at 72 h after initiation of antifungal treatment. Multifocal *Candida* colonization, preemptive treatment, and mechanical ventilation were associated with significantly lower rate of de-escalation (Tables 1 and 2). By multivariate analysis, only mechanical ventilation was independently associated with de-escalation (OR 0.25 (95% CI 0.08–0.74), *p *= 0.023; Hosmer–Lemeshow goodness-of-fit test, *p *= 0.95, *c*-statistics 0.85).

**Table 2:** Patient characteristics during ICU stay

Characteristics	De-escalation		*p*	OR [95% CI]
	Yes (*n *= 38)	No (*n *= 152)		
Multifocal colonization	19 (50)	115 (76)	0.002	0.32 [0.15–0.67]
Negative yeast samples	16 (42)	30 (20)	0.004	2.95 [1.4–6.3]
Empirical antifungal treatment	12 (32)	68 (45)	0.14	
Preemptive antifungal treatment	4 (11)	48 (32)	0.008	0.26 [0.09–0.78]
Targeted antifungal treatment	10 (26)	36 (24)	0.73	
Proven bacterial infection	10 (26)	0 (0)	< 0.001	NA
Apyrexia > 72 h	37 (97)	123 (81)	0.013	8.7 [1.2–66]
Catecholamine withdrawal at 72 h	29 (76)	89 (59)	0.026	2.57 [1.1–5.98]
Mechanical ventilation	30 (79)	142 (93)	0.006	0.26 [0.09–0.73]
Antibiotic treatment	38 (100)	150 (99)	0.96	
Total parenteral nutrition	18 (47)	81 (53)	0.51	
Surgery	21 (55)	76 (50)	0.56	
Renal replacement therapy	21 (55)	76 (50)	0.56	
Shock	31 (82)	121 (80)	0.79	

Data are N (%)

*CI* confidence interval, *OR* odds ratio

### Impact of de-escalation on outcomes

There was no significant impact of de-escalation on ICU length of stay, duration of mechanical ventilation, ICU mortality, 30-day mortality, or 1-year mortality rate (Table 3). In multivariable Cox proportional hazards model, only SAPS II and catecholamines withdrawal at day 3 were independently associated with ICU mortality, even when de-escalation was forced in the model (Table 4).

**Table 3:** Impact of de-escalation on outcome

Characteristics	De-escalation		*p*
	Yes (*n *= 38)	No (*n *= 152)	
Length of ICU stay	25 [14–40]	25 [14–40]	0.99
Duration of mechanical ventilation	22 [5–31]	20 [10–35]	0.43
Total duration of antifungal treatment	6 [5–18]	13 [7–25]	0.023
ICU mortality	17 (45)	89 (59)	0.13
30-day mortality	9 (24)	56 (37)	0.13
1-year mortality	21 (55)	97 (64)	0.33

Data are *N* (%), or median (interquartile range)

**Table 4:** Factors associated with ICU mortality by Cox proportional hazards models

Factors	Univariate analysis		Multivariate analysis	
	HR (95% CI)	*p*	HR (95% CI)	*p*
At ICU admission				
SAPS II	1.01* (1.004–1.02)	0.005	1.01* (1–1.02)	0.040
Surgical patients	0.46 (0.3–0.69)	< 0.001	–	–
ARDS	1.81 (1.22–2.68)	0.003	–	–
During ICU stay				
Renal replacement therapy	1.64 (1.1–2.47)	0.018	–	–
Preemptive antifungal treatment	0.50 (0.32–0.80)	0.004	–	–
Apyrexia > 72 h	0.36 (0.23–0.57)	< 0.001	–	–
Catecholamine withdrawal at 72 h	0.35 (0.27–0.52)	< 0.001	0.47 (0.29–0.76)	0.002
De-escalation of antifungal treatment**	0.75 (0.44–1.26)	0.28	–	–

*ICU* intensive care medicine, *SAPS* simplified acute physiology score, *ARDS* acute respiratory distress syndrome

* Per point of SAPS II; *HR* hazard ratio, *CI* confidence interval

** De-escalation of antifungal treatment was forced in the final Cox model

## Discussion

Our results suggest that de-escalation is performed in 20% of critically ill patients receiving empirical, preemptive, or targeted antifungal treatment for suspected or proven invasive *Candida* infection. Mechanical ventilation was the only factor independently associated with lower rates of de-escalation of antifungal treatment. No negative impact of de-escalation was found on duration of mechanical ventilation, ICU length of stay, ICU mortality, 28-day mortality, or 1-year mortality rates.

The incidence of de-escalation of antifungal treatment in our study is in line with that reported recently by Bailly et al. (22%) [26]. In another recent retrospective study performed in 262 critically ill patients receiving empirical or targeted antifungal treatment, the incidence of de-escalation was lower at 10% [29]. Azoulay et al. performed a large multicenter cross-sectional one-day study to determine the incidence of ICU patients without documented antifungal infection who receive antifungals. Antifungal treatment was used in 154 (7.5%) of study patients, including 100 (65%) patients without documented fungal infection. These results suggest that de-escalation of antifungal treatment could probably be performed in a larger proportion of critically ill patients.

Invasive mechanical ventilation was the only factor independently associated with lower rates of de-escalation of antifungal treatment. This result could be related to the higher severity of patients receiving invasive mechanical ventilation, which might have prevented attending physicians from de-escalating antifungal treatment. The superiority of echinocandins over fluconazole has been demonstrated in a single head-to-head clinical trial of anidulafungin that showed significantly better overall response rates (76 vs. 60%; *p* = 0.01) [30]. A further post hoc analysis showed better global responses (70.8 vs. 54.1%) and reduced 14-day all-cause mortality (10.1 vs. 20.3%, *p* = 0.08) in critically ill patients [31]. However, a recent large multicenter observational study, using a propensity-score derived analysis, did not report increased mortality using fluconazole as empirical or targeted treatment, as compared with echinocandins, in adult patients with candidemia [32]. Similar results were also reported in the subgroup of patients with sepsis or septic shock.

Recent observational and randomized controlled studies have questioned the beneficial effects of empirical antifungal treatment on mortality, even in patients in septic shock with high colonization index [33, 34]. However, the randomized controlled EMPIRICUS trial found significantly reduced rate of invasive candidiasis in patients who received micafungin, as compared with those who received placebo [34]. Our results suggest that de-escalation of antifungal treatment is safe. Overall, our results confirm previous studies, suggesting that early de-escalation to azole is possible and safe. For proven invasive *Candida* infection, three studies reported that de-escalation is safe in *Candida spp.* fluconazole-sensitive infections [24, 25, 35]. Bailly et al. [26] also reported that de-escalation could be safely performed in critically ill patients, as no negative impact was found on ICU mortality, duration of mechanical ventilation, or length of ICU stay. In addition, antifungal de-escalation was associated with significant decrease in the antifungal consumption, which might be helpful in reducing toxicity, drug interaction, fungal resistance, and cost [36]. A recent randomized controlled trial aimed to determine the usefulness of fungal biomarkers in early discontinuation of empirical antifungal treatment [37]. Patients were randomized to receive routine care (control group) or biomarker-based strategy (intervention group), in which a recommendation was given based on (1,3)-*β*-d-glucan, mannan, and anti-mannan serum assays performed on day 0 and day 4. The percentage of patients with early discontinuation of empirical antifungal treatment was significantly higher in intervention, compared with control group (54 vs. 2%, *p* < 0.0001), with no negative impact on mortality or morbidity. However, this open-label study was performed in a single center, and patients with immunosuppression were excluded. Therefore, further randomized controlled trials are needed to confirm these results.

Our study has some limitations. First, it was a retrospective study performed in a single center. Therefore, our results could not be generalized and further prospective multicenter studies are needed to confirm these findings. Second, the number of patients with de-escalation was relatively low. Therefore, analysis of subgroups with early stop, or reduction in antifungal spectrum was not possible. Third, potential benefit of de-escalation of antifungal treatment on cost was not evaluated. However, given the significant reduction in duration of antifungal treatment of 6 days in patients with de-escalation, compared with those with no de-escalation, a lower cost could be expected in these patients. Finally, no data were collected on dose or duration of corticosteroids. Corticosteroids use is a risk factor for invasive Candida infection and could impact on prognosis of patients with these infections. However, it is unlikely that corticosteroids have influenced de-escalation of antifungal treatment.

## Conclusions

De-escalation was performed in 20% of patients receiving systemic antifungals for suspected invasive *Candida* infection. Invasive mechanical ventilation was independently associated with reduced de-escalation of antifungal treatment. De-escalation was associated with decreased antifungal treatment duration. De-escalation of antifungal treatment seems to be feasible and safe in critically ill patients. However, further large prospective studies are required to confirm these findings.
